# Effects of Glutamine Supplementation on Atlantic Salmon *Salmo salar* Metabolic Performance at High Temperatures

**DOI:** 10.1155/anu/6632942

**Published:** 2024-11-28

**Authors:** Barbara Nuic, Alyssa Bowden, Artur Rombenso, Michael Salini, Matthew K. Jago, Richard Smullen, Craig E. Franklin, Rebecca L. Cramp

**Affiliations:** ^1^School of the Environment, The University of Queensland, Brisbane 4072, Queensland, Australia; ^2^CSIRO, Agriculture and Food, Livestock and Aquaculture Program, Bribie Island Research Centre, Woorim 4507, Queensland, Australia; ^3^Nutrition and Seafood Laboratory (NuSea.Lab), School of Life and Environmental Sciences, Deakin University, Queenscliff, Victoria, Australia; ^4^Ridley AgriProducts Pty Ltd., Narangba, Queensland, Australia

**Keywords:** amino acid, digestion, oxygen consumption rate, physiology, specific dynamic action, thermal stress

## Abstract

Atlantic salmon are one of the most important fish species in global aquaculture production. However, temperature increases attributed to climatic events impair the production of Atlantic salmon during summer. Additionally, the nutritional requirements for this species when reared under elevated temperatures require elucidation. To address this gap, a feeding trial was conducted to investigate the effect of glutamine supplementation—a functional amino acid (AA) important for energy production and gut health—on the growth, metabolism, gut morphology, antioxidant capacity and thermal tolerance of Atlantic salmon parr at elevated temperatures (22°C). Atlantic salmon were pair-fed three isoenergetic diets: a control diet (D1, no addition of glutamine), D2 (7% glutamine supplementation with other dietary AA levels reduced—isonitrogenous to D1) and D3 (6% glutamine and with the same AA profile as D1). Metabolic rate measurements and sampling commenced after 7.5 weeks on diets and 3 weeks of exposure to 22°C. Glutamine supplementation (D2 and D3) did not affect specific growth rate (SGR), condition factor, relative gut mass or carcass composition despite fish fed D3 having increased pyloric caeca fold height (hF). Resting, maximum and digestive metabolic rates were also unaffected by glutamine supplementation. Contrary to findings in other fish species at optimum temperatures, this study showed that glutamine supplementation did not improve the growth performance of Atlantic salmon parr at elevated temperatures despite enhancing pyloric caeca surface area.

## 1. Introduction

Atlantic salmon (*Salmo salar* L.) are an important food source, with global farmed fish totalling 2.7 million metric tons annually [1]. However, rising sea surface temperatures significantly impact Atlantic salmon production during the summer grow-out period, when elevated temperatures lead to decreased feed intake, growth and occasionally mortality [2, 3]. While there is a growing interest in diet interventions to mitigate thermal stress, there is still little understanding of the nutritional requirements and potential diet interventions to support fish health and growth at elevated temperatures [4, 5]. Numerous studies have explored the nutritional requirements of this species in culture; however, they have mostly been conducted under optimum temperatures for growth [6–12]. Thus, further studies are required to investigate whether diet interventions can mitigate stress and support growth in Atlantic salmon at high temperatures.

Glutamine is a functional amino acid (AA) due to its several roles in supporting tissue metabolism [13, 14]. This AA is a precursor of important metabolites that aid an organism's homeostasis, such as the antioxidant glutathione, and its supplementation can ameliorate oxidative damage caused by reactive oxygen species (ROS) [14–17], which tends to increase at elevated temperatures [18]. Excess oxidative damage can lead to cell death; hence, mitigating it could also potentially improve fish performance and thermal tolerance. Furthermore, growth is a result of a balance between protein synthesis and degradation, which relies on the adequate provision of AAs to meet metabolic demand [14]. Recent studies showed that glutamine plays a key role in the metabolism of Atlantic salmon at elevated temperatures [19, 20]. Thus, glutamine supplementation might be required to improve Atlantic salmon's overall health and growth, particularly at elevated temperatures.

Glutamine has been shown to play a key role in energy metabolism [13, 14, 21]. Glutamine is first catabolized to glutamate, which then can be catabolized to α-ketoglutarate (α-KG). α-KG is a key metabolite that fuels the citric acid cycle, a central pathway in adenosine triphosphate (ATP) production. Studies have shown that glutamine and glutamate are the preferred substrates for ATP production in various fish tissues, including skeletal muscle, which accounts for a large part of an animal's energy expenditure, as well as the intestine [13, 22]. The role of glutamine as an energy substrate for the intestine cells—enterocytes—which are responsible for nutrient absorption, likely supports the increase in the gut surface area observed in several fish species [23, 24], which is essential for enhancing digestive efficiency and maximal growth. While other AAs, such as branched-chain AAs (e.g. isoleucine, leucine and valine), can also be metabolized into α-KG to support ATP synthesis, this pathway would be inefficient, as these AAs play a critical role in protein synthesis and growth [25]. Thus, by directly supporting ATP production, glutamine may spare other AAs from being utilized for energy, allowing them to be redirected toward growth processes [6, 26, 27].

The provision of an adequate amount of the preferential energy substrate becomes particularly relevant at elevated temperatures, which heightens the energy demands of fish. Indeed, studies have demonstrated that the cost of baseline metabolism (standard metabolic rate [SMR]) and digestion, absorption and assimilation of a meal (specific dynamic action [SDA]) of hapuku (*Polyprion oxygeneios*) and Atlantic salmon increase with temperature [28, 29]. The cost of digestion is unavoidable to achieve growth, and it can consume a significant portion of the energy budget (aerobic scope [AS]), which for some species such as Atlantic salmon is worsened at high temperatures due to a decrease in the AS combined with increased SDA [29, 30]. The restricted AS can result in aerobic demands such as digestion and growth being prioritized over one another [31–33]. However, diet composition can affect the energy required to digest a meal, and its manipulation can be used as a strategy to reduce the constraint of SDA on the AS [30, 34–36]. Fine-tuning AA balance in the diet could reduce digestion costs and constraints on AS by preventing costly secondary biochemical reactions to balance AAs in the organism to maintain homeostasis [31, 37–40]. Furthermore, the potential increased gut surface area and the consequent enhanced gut function could reduce the SDA [41]. Thus, there is increasing evidence that glutamine requirement escalates with temperature, and its supplementation has the potential to support fish's increased energy demand, digestion and associated costs at elevated temperatures. However, the impact of diet on the digestion energetics and its influence on growth are still poorly understood; as to date, only two studies have measured the SDA in this species [29, 42].

The aim of the present study was to investigate the effects of glutamine supplementation on Atlantic salmon metabolism at elevated temperatures. We hypothesized that (1) glutamine-supplemented diet will result in decreased cost of digestion (SDA), resulting in more energy available for growth; (2) glutamine supplementation will promote enterocyte proliferation (intestine epithelial cells), improving the gut surface area and contributing to improved gut health and digestive efficiency; and (3) improved antioxidant capacity will reduce oxidative damage and enhance thermal tolerance.

## 2. Methods

All experimental procedures were carried out with the approval of The University of Queensland Animal Ethics Committee (Ethics 2022/AE000299).

### 2.1. Experimental Diets

Diets were formulated and manufactured at Bribie Island Research Centre (Commonwealth Scientific and Industrial Research Organisation [CSIRO], Australia) with a diet mash provided by Ridley Aquafeeds (Narangba, QLD, Australia) as extruded pellets. Diets were extruded through a laboratory-scale 24-mm twin-screw extruder (MPF24:25, Baker Perkins, Peterborough, UK), with intermeshing, corotating screws, and pellets were cut to 4.5-mm lengths at the die face with a four-blade variable speed cutter. The pellets were then dried at 60°C for 24 h. Following drying, the diets were vacuum infused with a specific allocation of oil. The three experimental diets were isoenergetic with the control diet (D1) containing 7% glutamine + glutamic acid. L-Glutamine (Bulk Nutrients Pure Supplements, Australia) was added to diets D2 and D3. D2 was supplemented with 7% glutamine (total of 13% glutamine + glutamic acid) with a concomitant reduction in other AAs so that the diet was isonitrogenous with D1, and D3 was supplemented with 6% glutamine (total of 13% glutamine + glutamic acid) and no reduction in the other AA (higher overall AA content) with reduced lipid content to keep it isoenergetic to D1 (Tables 1 and 2).

Diet D2 was formulated to be isonitrogenous to D1 to investigate the effect of glutamine supplementation without the compounding effect of increased total protein levels. Diet D3 had an AA profile similar to D1 to investigate the effect of glutamine supplementation without the potential impact that reducing other AAs may have on fish performance. Overall, D3 had a slightly higher protein content than D2 (Table 2). Diets were stored at −20 and 4°C prior to feeding.

### 2.2. Fish Husbandry and Growth Trial

Atlantic salmon parr (*S. salar*, *n* = 250) were sourced from Forest Home Hatchery (Huon Aquaculture), Australia, and transported to The University of Queensland in oxygenated transport bags placed in foam boxes with gel coolers. Then, fish were randomly redistributed between 15 tanks (40 L, 60 × 25 × 30 cm) across two aquarium systems at a density of 15–16 fish tank^−1^ to recover from transport. Each freshwater recirculating system (1000 L) had a partial flow-through with filtered fresh water, exchanging 5%–10% of the water daily. Water was treated with mechanical and biological filtration and ultraviolet (UV) sterilization, and water quality was maintained at pH ~7.5, total ammonia nitrogen <0.5 mg L^−1^, air saturation >90% and at 15°C with chillers (Teco TK 2000, Italy). Fish were given 3 weeks to acclimate to the systems, fed to satiation once daily (Skretting 2.3 mm Nutra RC) and kept under 12 h light:12 h dark throughout the experiment.

Three weeks after transport, fish were fasted for 48 h and lightly anaesthetized with (benzocaine, 100 mg L^−1^), and individual body mass (g) and length (cm) were measured. Fish (*n* = 210) were then graded and redistributed across 15 tanks, five replicate tanks per diet, at a density of 14 fish per tank, to equalize tank biomass (266 ± 6 g, mean ± standard deviation [SD]) and average fish mass (19.0 ± 2.7 g, mean ± SD) between tanks. After 1 to 2 days of recovery and fed once a day, fish were fasted for 24 h, lightly anesthetized with benzocaine (100 mg L^−1^), tagged with visible implant elastomer (Northwest Marine Technology, USA) below their dorsal fin and returned to tanks to track individual growth (Table 3). Tagging occurred across 2 days.

**Figure 1:**
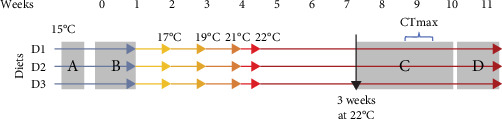
Experimental timeline for Atlantic salmon parr fed three isoenergetic diets (D1, D2 and D3). Weeks are relative to time on experimental diets. (A) Morphometrics and tagging. (B) Feeding of experimental diets to satiation to determine pair-feeding regime. Fish were pair-fed experimental diets from week 1. (C) Measurement of metabolic rate metrics, CTmax and carcass sampled for composition analysis. (D) Tissue sampling for histology and antioxidant analysis. Growth metrics were calculated from fish subsampled from C and D.

Four days after tagging, feeding of the experimental diets commenced (Tables 1 and 2). Fish were fed once a day between 9:00 and 10:00 h to satiation for a week to determine the feeding rate. Daily feed consumption was estimated based on the amount offered (preweighed) less any uneaten pellets. Uneaten pellets were counted ~20 min after the food was offered, and the total uneaten food was estimated based on average pellet mass (±0.01 g). The average daily feed intake was calculated for that week, and the feeding rate for all tanks was determined to be 85% of the lowest tank feed intake. This rate was readjusted weekly based on fish feeding response and an estimated feed conversion ratio (FCR) of 1. The feeding rate ranged from 1% to 1.3% body mass (BM) during the growth trial. After determining the feeding rate, the temperature was increased by 2°C per week up to 22°C by three thermostatically controlled stick heaters placed in each sump (Bioescape Tropic 300 W), and fish were acclimated for at least 3 weeks before measurements (Figure 1).

The final mass, length, liver and whole gut mass (the oesophagus to the posterior intestine without visceral fat) were measured in a subset of fish that were either fasted for ~47 h or 2–6 h postfeeding between 7.5 and 11 weeks on the experimental diet (equivalent to 3–7 weeks of exposure to 22°C, Figure 1) and used to calculate growth metrics (calculations described in the Table 3 footnote).

### 2.3. Metabolic Rate Measurements (SDA Parameters, SMR, Maximum Metabolic Rate [MMR] and AS)

Oxygen uptake rates (MO_2_) were recorded in 15–16 fish per diet across eight runs (over the course of 2.5 weeks) using intermittent flow-through respirometry [44], and measurements commenced after 3 weeks of exposure to 22°C (equivalent to ~7.5 weeks on experimental diets, Figure 1). Eight custom-made acrylic chambers (2.2 L) were submerged in pairs in 30-L opaque water baths. Each chamber had two pumps: one for water recirculation within the chamber (Aqua bee UP 300, 300 L h^−1^, Germany) that ran continuously and contained a fibre optic oxygen probe connected to a four-channel Fibreoptic Oxygen Meter (PyroScience, FireSting O2 FSO2-C4 and FSO2-x, Germany). The oxygen meter interfaced with the associated software Pyro Oxygen Logger (PyroScience, Germany), which recorded % air saturation in the chamber every second. The second pump (Aqua Zonic, Sumo G2-1 Amphibious pump 1200 L h^−1^, Singapore) was controlled by a custom-made timer (Axion, Australia) to flush chambers in cycles (4 min closed, 5 min flushing). Each water bath was equipped with air stones to maintain aquatic oxygen levels >80% air saturation, a chiller (Teco TK 2000, Ravenna, Italy) and a heater (Bioescape Tropic 300 W) to maintain water temperatures at 22 ± 0.5°C.

For MO_2_ measurements, up to eight fish (1–2 fish per tank) were netted from their experimental tank after feeding, anaesthetized, tag identified, weighed and individually transferred to empty holding tanks to fast for around 48 h. The holding tanks were between two other tanks containing conspecifics with glass walls to promote a feeding response since Atlantic salmon are communal feeders. One holding tank at a time was offered pellets of their respective diet simultaneously with neighbour tanks to instigate feeding until they ate a full ration (~5 min; 0.5% BM). Feed intake was recorded by counting the observed pellets ingested with a clicker. Fish that ate their complete ration were netted, placed in a small container with water to minimize stress due to air exposure and inserted in respirometry chambers through an opening at the top (<1 min per transfer). Then, the chamber was sealed with a rubber stopper and covered with opaque bubble wrap. Fish that failed to eat the full ration were returned to their experimental tanks.

Oxygen uptake rate was recorded for around 44 h and used to calculate SDA and SMR. Then, each fish was transferred from the chambers to a bucket with aerated water at 22°C, chased for 3 min by hand or net and then transferred back to the chambers to record the MMR. The final MMR was calculated based on the highest MO_2_ reached either postswimming or during SDA measurements. Next, fish were euthanized by anaesthesia overdose (benzocaine, 250 mg L^−1^), and the gut was dissected to confirm the end of digestion.

### 2.4. Metabolic Rate Calculation

Fish MO_2_ (mg O_2_ h^−1^) was calculated as the slope of the decline in oxygen concentration in the chambers during the closed phase of the cycles. Specifically,

  $$\begin{array}{c} {\text{MO}}_{2}=-1\times \Delta O_{2}\times V\times \beta O_{2}. \end{array}$$

ΔO_2_ is the rate of change of oxygen saturation within the respirometer (as % air saturation per hour), V is the volume of the respirometer minus the mass of the fish (assuming a density of 1 g mL^−1^), and *β*O_2_ is the solubility of oxygen in freshwater at 22°C [45].

The slopes were extracted using LabChart (v8.1.17), and MO_2_ was calculated on Microsoft Excel (version 16.65) for every closed cycle—every 10 min. Then, SMR and SDA metrics (described in Table 4) were calculated in R (v 4.2.1) with the ‘Fish MO2' package [46, 47]. A 10 h period was selected (from 19:00 to 5:00 h next day, ~34–44 h postfeeding) to determine SMR. This time was selected by plotting MO_2_ postfeeding on Excel to observe when MO_2_ plateaued (i.e. indicating the completion of the SDA response). SDA parameters were calculated with the function ‘calcSDA' (tau = 0.2, lambda = 24, tolerance fit = SMR ± 8%). The first 3 h of MO_2_ was estimated based on a linear regression from 3 h postfeeding to time 0 h (first measurement) to avoid overestimation due to stress caused by handling. This time was determined based on previous experiments and visual assessment of SDA plots [29]. Background respiration was measured for 30–60 min prior to transferring fish to chambers and was assumed to remain constant over the measurement period and was accounted for within MO_2_ calculations.

After calculations, SDA plots were generated with the ‘fishMO2' package to assess the individual's SDA curve variation. Seven individuals were excluded due to overestimation of SMR and, thus, SDA (i.e. high MO_2_ level throughout measurements potentially due to stress in chambers, which prevented the estimation of SMR and SDA). Also, six fish were excluded due to the presence of residual food in the stomach and midintestine despite the apparent completion of the postprandial metabolic response. Thus, 13 fish in total were excluded (omitted: 3–6 diet^−1^, final *n* = 10–13 diet^−1^).

### 2.5. Antioxidant Capacity and Oxidative Damage

To investigate the potential of glutamine supplementation to enhance antioxidant capacity and reduce oxidative damage, liver and gut sections were sampled in a subset of fish (*n* = 10–11 diet^−1^) after 10 weeks on the experimental diets (equivalent to around 6 weeks at 22°C, Figure 1). Fish were fasted for ~47 h, netted (2–3 tank^−1^) and euthanized with benzocaine (250 mg L^−1^) and then weighed (g), measured (cm) and tag identified. Next, the whole gut was dissected and weighed, and the midintestine and 1 cm of the pyloric caeca were removed and fixed for subsequent histological analysis (see below). The remaining pyloric caeca was frozen in dry ice and stored at −80°C for antioxidant analysis.

Total glutathione (TGSH) was quantified spectrophotometrically (DTX880, Beckman Coulter, California, USA) by enzymatic recycling assay following an adapted protocol from Massarsky, Kozal and Di Giulio [48]. Briefly, liver and pyloric caeca tissues were homogenized (1:100 w:v) in 5% sulfosalicylic acid for 1 min at 30 HZ (TissueLyser II, QIAGEN, Netherlands). Then, samples were centrifuged for 5 min at 5000× *g* at 4°C. Next, the supernatant was aliquoted to perform the assay immediately. Seven standards were prepared by dissolving reduced glutathione (G4251, Sigma–Aldrich, USA) in 5% sulfosalicylic acid at concentrations from 0 (control) to 100 μM. Measurements were made in a 96-well microplate in triplicates by adding 10 μL of supernatant or standard, 200 μL of assay solution (0.7 mM 6,6′-dinitro-3,3′-dithiodibenzoic acid [DTNB] and 0.3 mM nicotinamide adenine dinucleotide phosphate [NADPH] in 100 mM potassium phosphate buffer) and 10 μL of glutathione reductase (2 U/mL) into each well. Sample concentrations were determined through the rate of change in absorbance at 405 nm over 8–14 min that corresponds to the linear sections of the reaction (*R*^2^ > 0.98). Sample values were calibrated based on the on-plate standard curve. TGSH concentration (nmol mg tissue^−1^) was normalized to tissue mass.

Lipid peroxidation was measured as malondialdehyde (MDA) and quantified with a commercial assay kit (MAK085, Sigma–Aldrich) to determine the extent of oxidative damage in the liver and pyloric caeca.

### 2.6. Intestine Morphology and Analysis

The pyloric caeca and midintestine were sampled from the same subset of fish as above (*n* = 10 per diet). Around 1-cm pyloric caeca and midintestine sections were fixed in 10% neutral buffered formalin for 48 h and then transferred to 70% ethanol for storage until processing. Tissues were dehydrated in an increasing ethanol series (from 70% to 100%) and xylene and then submerged in paraffin wax in a tissue processor (Leica TP1020, Germany). Tissues were mounted in cassettes and covered with paraffin in the embedding working station (HistoStar, Thermo Fisher, USA). Tissues were sectioned transversely with a microtome (RM2235, Leica, Germany), and two 4-µm sections were collected from each block onto slides. Then, the slides were stained with haematoxylin and eosin. Slides were imaged in a light microscope (Zeiss Axio, Germany) with a magnification of 10 µm and equipped with a camera (Zeiss Axiocam 506, Germany) with software Zeiss ZEN (Zen 2 Pro Software, Zeiss, Germany). Morphometric measurements were taken in 8–10 random folds per fish which the central canal (lamina propria) was not fragmented. In each fold, fold height (hF) and two random enterocyte heights (hEs) (total of 16–20 per fish) were measured.

### 2.7. Critical Thermal Maximum (CTmax)

An acute thermal tolerance test was performed in a subset of fish (*n* = 12 diet^−1^) to investigate the effect of glutamine supplementation on acute thermal tolerance. CTmax tests were performed after 5 weeks of acclimation at 22°C (equivalent to 9 weeks on the experimental diets, Figure 1). Tests were run across 3 days, with two runs per day (total of six runs). Briefly, fish were fed as usual at 9:00 h. The first run started 1 h postfeeding and second run started 2.5 h postfeeding. For each run, one to three fish per diet (total of five to seven fish) were netted from random experimental tanks, transferred to a bucket to identify tag and transferred to a glass test tank (21 L, at 22°C ± 0.5°C). The tank had air stones connected to an air pump (Aquaworld, AW-404), two heaters (Bioescape Tropic 300 W; Aquazonic 300 W), one circulation pump (Fluval Sea CP2 100 L, 1600LPH) and a digital thermometer (TP101, TENMA, Japan, accuracy of ± 0.1°C). After 1 h adjusting to the test tank, the water temperature was increased at 0.1°C min^−1^ until the fish lost equilibrium (LOE; lost capacity to maintain dorsal–ventral position). Then, fish were removed and euthanized, weighed (g) and measured (length, cm) and tag identified. Next, fish were then dissected to confirm the presence of food in the gut. Then, the viscera was removed, and the carcass was weighed and frozen at −20°C for later composition analysis.

### 2.8. Diet and Carcass Composition

Diet and carcass composition were determined by established methodology (Association of Official Analytical Collaboration [AOAC] [49], from a random subset of 30 fish used for CTmax testing. Carcasses were pooled within diets by homogenization of three to four fish for proximate analyses (final *n* = 3 diet^−1^ after pooling individuals). First, carcass samples were freeze-dried (Alpha 1–4, Martin Christ, Germany) and homogenized. Dry matter was determined gravimetrically in carcass and diets by drying samples at 105°C. Next, carcass and diet samples were ashed for 24 h at 550°C in a muffle furnace to determine gross ash. Total lipid was determined gravimetrically by methanol and chloroform extraction. Total nitrogen was quantified in an elemental analyser (CHNS-O Flash 2000, Thermo Scientific, USA), and crude protein was determined by multiplying nitrogen content by 6.25. Gross energy was determined by a bomb calorimeter (Par Instrument Company, Moline, USA).

Diet AA composition was determined using a high-performance reverse-phase liquid chromatography–mass spectrometry (LC–MS) (LCMS 8030, Shimadzu Corporation, Japan) as detailed by Truong et al. [50]. Briefly, samples were digested in 0.1 N hydrochloric acid and diluted with 18 Ω water and filtered, and aliquots were derivatized and tagged with 6-aminoquinolyl-N-hydroxysuccinimidyl carbamate (AQC). In this protocol, glutamine is deaminated during acid hydrolysis, and consequently, glutamic acid reported in Table 2 represents the total glutamine and glutamic acid present in diets [43].

### 2.9. Statistical Analyses

Analyses were performed in the statistical program R (version 4.1.2, R Core Team [51]) using the RStudio interface (version ‘Spotted Wakerobin', 2022). Data are presented as means ± SD unless otherwise stated. Response variables were checked for normal distribution and equal variance with functions ‘shapiro.test' and ‘leveneTest', respectively (package: ‘car' [52]). Metrics that did not meet the criteria were log-transformed (condition factor, SDA magnitude, SDA cost, final mass, hE from the pyloric caeca and mid intestine). Model selection for each parameter was done by comparing models by analysis of variance (ANOVA) and reducing one fixed or random effect at a time until the best model was found according to the lowest Akaike information criterion (AIC) and Bayesian information criterion (BIC) values.

The effect of diet on initial fish mass and length; mass and length from the subset of fish used in metabolic rate measurements, feed intake and energy of meal ingested for SDA measurement; carcass mass and length from the subset of fish used for CTmax, carcass composition, TGSH and oxidative damage metric (MDA); and metabolic rate metrics (described on Table 4) were investigated by one-way ANOVA. For TGSH and MDA, a model was fitted for each tissue (liver or pyloric caeca). Also, one outlier was excluded from TGSH data for D3 liver samples based on boxplot observation (i.e. the excluded value was below the first quantile – 1.5 × interquartile range).

The effect of diet on growth metrics (final mass and length, body mass gained [BMG], condition factor, hepatosomatic index, relative gut mass and specific growth rate [SGR]), CTmax and histological parameters (midintestine and pyloric caeca fold and hE) was analysed with individual linear mixed-effect models using the R function ‘lmer' from the *lmerTest* package [53]. For growth metrics, diet was treated as a fixed effect and days on the diets as a random effect. Days on the diets weres added as a random effect to account for the long sampling period, hence, potential influence of different time on the diets and exposure to elevated temperature. The SGR model had condition factor as a random effect to account for potential behavioural advantage in obtaining food, given that pair-feeding could have resulted in some individual's feed intake being lower than others. Thus, growth rates would be affected by the individual feed intake and not diet, which is not possible to measure directly. The condition factor model had tank ID as a random effect. The CTmax model had diet as a fixed effect, carcass mass as a covariate and date the test was conducted as a random effect. For histological parameters, hF or hE were treated as the response variable, diet as a fixed effect, mass as a covariate and fish ID as a random effect to account for repeated individual measurements.

*p*-Values were estimated with Type II Wald F tests with Kenward–Roger degrees of freedom analysis (package ‘car', function ‘Anova' [52]). Significance was accepted at *p*  < 0.05. Post hoc tests were estimated with marginal means with function ‘emmeans' where appropriate (package: emmeans [54]).

## 3. Results

### 3.1. Growth Parameters and Carcass Composition

There was no difference between treatments in initial body mass and length (Table 3). After 7.5–11 weeks on the experimental diets (three to seven at 22°C), no difference was found in the final mass, length, SGR, body mass gain, condition factor, hepatosomatic index or relative gut mass (Table 3).

Mass and length from the subset of fish used for carcass composition did not differ between treatments (Table 5). Furthermore, diet did not influence ash, total lipid, crude protein or energy content (Table 5).

### 3.2. Metabolic Rate

The body mass and length of the subset of fish used for oxygen uptake rate measurements did not differ between treatments (Table 6). Also, there was no significant difference in feed intake or energy consumed between treatments for metabolic rate measurements (range: 0.46–0.57% BM, Table 6).

**Figure 2:**
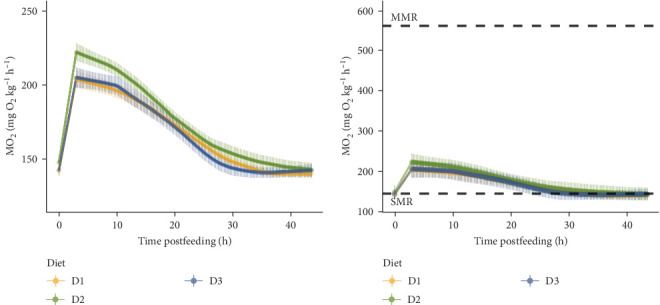
Oxygen uptake rate (MO_2_) postfeeding of Atlantic salmon parr at 22°C fed one of three isoenergetic experimental diets that differ in glutamine and total amino acid levels (D1, D2 and D3, *n* = 10 −13). (A) Dots and bars represent the mean and standard error (SE) of MO_2_ for every 10 min. Estimated mean standard metabolic rate (SMR) is indicated as the first MO_2_ value followed by a linear regression to estimate the first 3 h of oxygen uptake rate postfeeding. (B) Postfeeding MO_2_ represented in the context of the aerobic scope (AS). Dots represent mean and bars standard deviation (SD). Mean SMR and maximum metabolic rate (MMR) values between diet treatments are indicated by dashed black lines.

There was no effect of diet found on SMR or MMR nor AS (Table 6). Also, there was no difference between diet treatments on postfeeding metabolic rate metrics (SDA) (Table 6, Figure 2).

### 3.3. Antioxidant Capacity and Oxidative Damage

**Figure 3:**
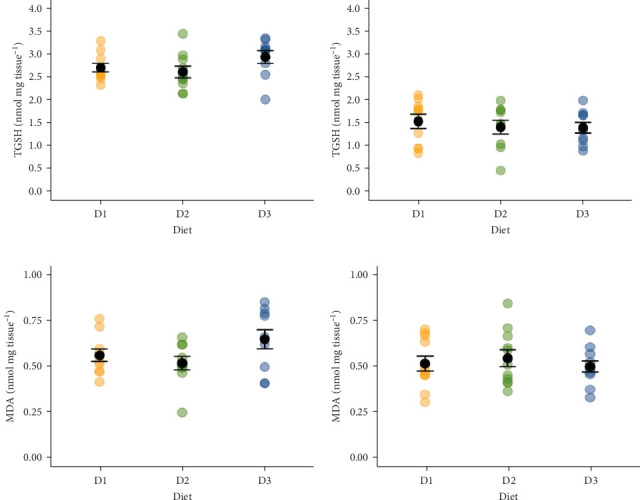
Antioxidant total glutathione (TGSH), in the liver (A, *n* = 9–10) and pyloric caeca (B, *n* = 10), and malondialdehyde (MDA) in the liver (C, *n* = 10) and pyloric caeca (D, *n* = 11) of Atlantic salmon parr fed three diets (D1, D2 and D3) following acclimation to 22°C. Black dots and bars represent mean ± standard error (SE). Raw data in yellow (D1), green (D2) and blue (D3).

There was no influence of diet on the TGSH in the liver (*F*_(2,26)_ = 1.855, *p* = 0.177, Figure 3A) and caeca (*F*_(2,27)_ = 0.305, *p* = 0.740, Figure 3B) of Atlantic salmon parr chronically exposed to 22°C. Also, MDA was measured as a proxy for oxidative damage through lipid peroxidation. Diet did not influence the MDA levels in the liver (*F*_(2,27)_ = 2.529, *p* = 0.098, Figure 3C) or pyloric caeca (*F*_(2,27)_ = 0.324, *p* = 0.726, Figure 3D).

### 3.4. Gut Macromorphology

Glutamine with an overall AA increase (diet D3) significantly affected the hF in the pyloric caeca but had no effect on hE (Table 7). Specifically, hF increased in fish fed diet D3 compared to diet D1 (pairwise _D1–D3_: *p* = 0.027) but not in fish fed diet D2 (pairwise_D2–D3_: *p* = 0.750). There was no difference between diet treatments in hF and hE in the midintestine (Table 7).

### 3.5. Thermal Tolerance (CTmax)

**Figure 4:**
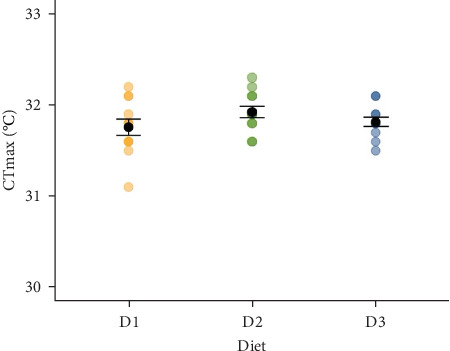
Critical thermal maximum (CTmax, *n* = 12 diet^−1^) of Atlantic salmon parr fed three diets (D1, D2, D3) following acclimation to 22°C. Black dots and bars represent mean ± SE. Raw data in yellow (D1), green (D2), and blue (D3).

The carcass mass (mean: 44.0 ± 7.5 g, *F*_(2,33)_ = 1.826, *p* = 0.177) and length (mean: 15.3 ± 0.7 cm, *F*_ (2,33)_ = 1.117, *p* = 0.347) of fish used for the CTmax test did not significantly differ between diet treatments. Also, CTmax was not influenced by diet treatment (*F*_(2,30.2)_ = 1.317, *p* = 0.293) or mass (*F*_1(30.3)_ = 2.875, *p* = 0.100, Figure 4).

## 4. Discussion

Adequate provision of dietary AAs is vital to maximize fish health and growth performance [6, 13, 22, 27, 55]. However, little is known about how dietary requirements change in animals under challenging thermal conditions such as those imposed by climate change. In the present study, we investigated the effects of glutamine supplementation considering its fundamental role in the energetics of several tissues and its potential to reduce the cost of digestion and to support overall fish health by enhancing antioxidant capacity and thermal tolerance. We found no difference in growth parameters, metabolic rate (fasted and post-feeding) or thermal tolerance of Atlantic salmon. However, we observed an improved gut macromorphology.

Glutamine supplementation does not appear to improve the growth of Atlantic salmon parr at elevated temperatures. This was evidenced by similar values of BMG, SGR and condition factor between the standard diet and those supplemented with glutamine. Although this finding is consistent with those of Caballero-Solares et al. [26] who also found no effect of glutamine supplementation for seabream (*Sparus aurata*), they contrast with the findings of several studies with other fish species that have reported a positive response to glutamine supplementation for growth [16, 56–61]. However, these studies were conducted at optimum temperature in contrast to the present study that was conducted at an elevated temperature, which could influence both how glutamine was utilized by the animal and its nutritive requirement.

The lack of growth difference between diets in Atlantic salmon parr is consistent with the fact that their energy budget—AS—and SDA response were similar across diet treatments. This suggests that there was no difference across diets in the amount of energy allocated to digestion and assimilation of a meal (SDA) and how much it limited the AS (as the proportion of AS occupied by Peak_net_) and, consequently, the amount of energy available for growth. It also suggests that the SDA response might be influenced more by the energy content of the meal than the total amount of AAs (total protein content) [62, 63]. Also, it could indicate that the cascade of protein synthesis and catabolism that accompanies feeding, which largely contributes to SDA [64], did not significantly differ between diets. This suggests that all diets, including the control D1, presented an AA profile that met the nutritive demands of Atlantic salmon parr at 22°C and, therefore, explained the lack of difference in the metabolic rate and growth rate across the diets.

Glutamine is the preferential energy substrate of intestinal epithelial cells [22]. We observed an increased intestinal surface area in fish fed glutamine with an increase in overall AAs (diet D3). Thus, it is possible that the additional glutamine was mostly utilized by the intestine, preventing it from reaching the plasma and being utilized as energy by other tissues [65], hence having little effect on fish overall energetics (SMR and AS) and growth. Curiously, this increased surface area did not impact the SDA (i.e. did not reduce digestion cost) as expected. The lack of difference in protein and lipid carcass composition further suggests that glutamine was not metabolized as the preferential energy substrate in other tissues, and consequently, it did not have an AA-sparing effect to improve protein deposition. Our finding contrasts with Caballero-Solares et al. [26], who observed improved protein retention in gilthead seabream fed glutamine despite not observing growth differences. Thus, contrary to our hypothesis, there was no evidence that glutamine supplementation alone (diet D2) or paired with an overall higher AA amount (diet D3) improved digestion or energy metabolism efficiency to allocate more energy for growth nor improve AA utilization for protein deposition. However, fish fed diet D3 experienced an increase in pyloric caeca surface area, which aligns with findings from previous studies that observed an enhancement in intestinal morphology of fish species fed diets supplemented with glutamine [23, 56, 66–68]. The pyloric caeca plays a major role in the digestion and absorption of nutrients in salmonids [69, 70], and enhanced surface area may be particularly beneficial to fish at elevated temperatures, as there is an increase in gut transit [71] which could reduce absorption efficiency. The increased gut surface area could significantly improve the digestion and absorption of nutrients in fish at elevated temperatures. The true benefit of the increased gut surface area might have been realized if growth rates at the highest temperature were measured for longer.

It is important to consider that, in the present study, fish were fed their complete ration once daily, and a high proportion of the glutamine in the test diets was supplemented as a free AA, which can be absorbed at a faster rate than glutamine in the protein-bound form [72, 73]. The benefits of glutamine and overall AA supplementation might have been effective if fish were fed in smaller and more frequent meals. For example, Yamada, Tanaka and Katayama [74] demonstrated that diets composed of free AAs were better utilized by carp (*Cyprinus carpio*) and translated into growth when feeding was partitioned into multiple meals. Also, the capacity for fish to efficiently utilize glutamine as an energy substrate is limited by the abundance and activity of enzymes such as glutaminase, glutamate dehydrogenase and glutamate transaminase [13, 21, 75, 76] which were not investigated in this study. Also, if glutamine is provided in excess of immediate requirements, surplus glutamine would be excreted, not stored [77]. Thus, Atlantic salmon parr fed diets D2 and D3 as a single meal might have elicited a brief glutamine peak in the plasma and tissues, surpassing the fish's immediate requirements and their capacity to metabolize it, partially wasting the glutamine supplemented.

Only two levels of glutamine supplementation were tested in the present study (control with no addition of glutamine and 7% and 6% supplementation for diets D2 and D3, respectively), and the optimum amount may fall below or beyond the tested levels. For example, a study with hybrid striped bass (*Morone chrysops* × *Morone saxatilis*) found improved growth with the addition of 1% of glutamine on the basal diet, but 2% exceeded the optimum requirement for this species [56]. Thus, the fine scale of the nutritional requirement for this AA and its interaction with the overall dietary AA profile may be explored in future studies particularly under thermal challenge. Still, comparing studies and determining the adequate glutamine requirement are challenging as many studies often only report free glutamine added and not the total amount present in the diet. This can vary substantially since glutamine is present in many aquafeed ingredients at different concentrations [13]. Furthermore, studies with Atlantic salmon smolt that investigated the addition of 0.75%–1.5% of glutamate to the basal diet (crude protein ranged from 35% to 53%)—an AA that is largely interchangeable with glutamine for many functions—also found no influence of the supplementation on growth [9, 11, 78]. Hence, the dose investigated in the present study might have been outside the optimum range for Atlantic salmon parr at 22°C. Alternatively, the inclusion of glutamine present in the control diet may have provided adequate glutamine which would suggest supplementation into this formulation may not have been required.

Chronic exposure to high temperatures can cause oxidative damage resulting from an imbalance between the production of ROS and the ability of antioxidants to neutralize them [79]. This excessive ROS can lead to thermal injury by increased denaturation of protein, lipids and DNA, resulting in poor thermal tolerance at high temperatures [80–82]. However, studies have demonstrated that diet manipulation favouring increased antioxidant capacity can improve thermal tolerance in some fish species [83–85]. CTmax is an acute test that represents the endpoint at which cumulative heat damage results in organism failure. This endpoint depends on time spent at temperatures at which there is a higher ‘rate of disruption' than the organism is capable of offsetting to maintain homeostasis [86]. Given that all fish were subjected to the same CTmax protocol (heating rate), it would be expected that increased antioxidant capacity would improve their repair capacity and delay heat injury accumulation, resulting in higher CTmax in fish fed diets supplemented with glutamine (D2 and D3). However, we observed no differences in upper thermal tolerance limits, suggesting glutamine supplementation did not aid acute thermal tolerance. Also, we observed no effect of diet on antioxidant capacity (TGSH) and MDA levels in the liver and pyloric caeca of fish chronically exposed to 22°C. This indicates that the chronic exposure might not have caused a significant increase in the oxidative damage in the tissues investigated; hence, no difference of antioxidant response would be expected between diets. However, we would still expect a delay in the CTmax if glutamine was aiding antioxidant capacity when acutely exposed to further thermal stress. Furthermore, the lack of difference between diets for acute thermal tolerance could be a further indication of the poor utilization of glutamine supplementation as a free AA and warrants further investigation. These findings contrast with other studies that have found a beneficial effect of dietary glutamine supplementation improved antioxidant capacity and reduced oxidative damage in several fish species [23, 56, 58, 60, 61]. Thus, there is no evidence of glutamine supplementation offsetting oxidative damage in acute and chronic heat exposure in Atlantic salmon parr.

## 5. Conclusion

Glutamine supplementation does not appear to affect postprandial energetics and the amount of energy available for growth in Atlantic salmon parr. However, it does seem to enhance the gut surface area. Despite these effects, we found no improvement in growth performance, thermal tolerance, antioxidant capacity or oxidative damage. It is possible that the levels of glutamine provided in our study either exceeded or did not reach the optimum levels for this species under chronic high-temperature. Additionally, the benefits of glutamine supplementation might have been attenuated due to the large amount supplemented as a readily absorbable free AA. Future studies should explore a range of supplementation levels and offer as multiple daily meals, to better mirror aquaculture practices and optimize the benefits of glutamine supplementation in fish nutrition and welfare.

## Figures and Tables

**Table 1:** Experimental diet formulation.

Ingredients (%)	D1	D2	D3
Plant-origin products	10	7	8
Animal-origin products	60	53	60
Wheat flour	11.9	13.7	9.5
Micronutrients	3.1	3.8	3.2
Oils	15	15.5	13.3
L-Glutamine	0	7	6

*Note:* The full ingredient composition is commercial-in-confidence. Ingredients were provided by Ridley Aquafeeds, Australia. Plant-origin products included lupin meal and wheat gluten. Animal-origin products included fish meal, blood meal and poultry protein concentrate. Micronutrients included amino acids, vitamin and mineral supplementation (proprietary formulation), health-promoting inclusions, choline chloride 70% and astaxanthin. Oil source was a mixture of fish oil (~20%) and poultry oil (~80%). D1 (control), diet containing 7% glutamic acid; D2, supplemented with 7% glutamine and with a concomitant reduction in other amino acids; D3, supplemented with 6% glutamine and no reduction in the other AA (higher overall AA content). L-Glutamine was obtained from Bulk Nutrients Pure Supplements, Australia.

**Table 2:** Analysed proximate and amino acid composition for the three experimental diets.

Diet	D1	D2	D3
Composition (% dry weight [DW], except dry matter)			
Dry matter	97.8	97.2	97.6
Crude protein (% DW)	55.4	59.8	60.9
Total protein (sum of amino acids excluding taurine g kg^−1^)	481.9	504.9	550.3
Total lipid (% DW)	26.2	23.7	21.7
Ash (% DW)	7.7	7.5	7.8
Gross energy (MJ/Kg)	24.5	24.1	24.5
Amino acid composition (g kg^−1^)			
Alanine	30.9	27.9	31.2
Arginine	28.2	24.9	28.2
Aspartic acid	45.8	41.4	47.3
Cysteine	4.9	4.2	4.9
Glutamic acid + glutamine^a^	70.3	131	132
Glycine	26.8	24.7	27.3
Histidine	14.3	11.9	13.1
Isoleucine	20.6	17.9	20.4
Leucine	40.8	36.9	41.7
Lysine	40.6	36.6	43.3
Methionine	26.7	30.7	27.7
Phenylalanine	23.4	20.8	23.7
Proline	21.8	19.0	22
Serine	21.2	18.8	21.3
Taurine	2.2	2.0	2.1
Threonine	21.7	19.6	22.4
Tyrosine	16.8	14.3	16.5
Valine	26.9	24.4	27.2
Sum of amino acids (excluding taurine g kg^−1^)	481.9	504.9	550.3

^a^Glutamine is deaminated during acid hydrolysis, and consequently, glutamic acid reported in Table 2 represents the total glutamine and glutamic acid present in diets [43].

**Table 3:** Morphometrics, specific growth rate (SGR), body mass gained (BMG), condition factor (K), hepatosomatic index (HSI) and relative gut mass (RGM) of Atlantic salmon parr fed three experimental diets. Values are in mean ± SD.

Diet	D1	D2	D3	*F*-value	*p*-Value
Initial (*n* = 70)					
Mass (g)	19.1 ± 2.7	18.7 ± 2.4	18.7 ± 3.1	*F*_(2,207)_ = 0.476	0.622
Length (cm)	11.5 ± 0.6	11.4 ± 0.5	11.4 ± 0.6	*F*_(2,207)_ = 0.345	0.591
Final (*n*)	49	51	51	—	—
Mass (g)	51.2 ± 8.2	51.3 ± 5.6	51.8 ± 6.8	*F*_(2,144)_ = 0.230	0.795
Length (cm)	15.5 ± 0.8	15.6 ± 0.6	15.6 ± 0.7	*F*_(2,144.7)_ = 0.507	0.603
SGR (% day^−1^)	1.34 ± 0.18	1.35 ± 0.18	1.38 ± 0.20	*F*_(1,147.3.3)_ = 0.782	0.460
BMG (%)	169.9 ± 34.9	172.9 ± 34.4	178.9 ± 40.6	*F*_(2,144.6)_ = 1.048	0.353
K	1.37 ± 0.11	1.34 ± 0.07	1.35 ± 0.08	*F*_(2,11.9)_ = 0.640	0.545
HSI (%)	0.81 ± 0.17	0.84 ± 0.14	0.78 ± 0.14	*F*_(2,143)_ = 2.103	0.126
RGM (%) (*n* = 25–27)	3.88 ± 0.40	3.92 ± 0.43	3.76 ± 0.50	*F*_(2,72.4)_ = 0.883	0.418

*Note:* Values are in mean ± standard deviation (SD). Fulton's condition factor (K) = (body mass (g))/length (cm)^3^) × 100. Specific growth rate (SGR) = [(ln final body mass − in initial body mass)/days growing] × 100. Body mass gain (BMG) = [(final body mass (g) − initial body mass (g))/initial body mass (g)] × 100. Relative gut mass (RGM) = (gut mass/final body mass) × 100. Hepatic somatic index (HSI) = (liver mass/final body mass) × 100.

**Table 4:** Metabolic rate variable abbreviations and definitions.

Variable abbreviation	Definition
SMR	Minimum oxygen uptake at nondigestive resting state
MMR	Maximum oxygen uptake value postfeeding or postexercise
AS	Aerobic scope = MMR − SMR
SDA_peak_	Maximum oxygen uptake value postfeeding
Peak_net_	SDA_peak_ − SMR
*T*_peak_ (h)	Hours postfeeding when SDA_peak_ occurs
SDA_magnitude_	Total oxygen uptake postfeeding
SDA_cost_ (kJ.kg^−1^)	Total oxygen uptake postfeeding converted to energy assuming 1 g O_2_ = 14 kJ
SDA_duration_ (h)	Time postfeeding which oxygen uptake returns to SMR± 8%
SDA_scope_	The ratio between SDA_peak_ and SMR
SDA coefficient (%)	Energy spent to digest the meal relative to the energy of the meal.

*Note:* All MO_2_ values are in mg O_2_ Kg^−1^ h^−1^ except for SDA magnitude (mg O_2_ kg^−1^).

Abbreviations: AS, aerobic scope; MMR, maximum metabolic rate; SDA, specific dynamic action; SMR, standard metabolic rate.

**Table 5:** Carcass mass and length (*n* = 10 diet^−1^) of Atlantic salmon parr acclimated at 22°C and fed three experimental diets (D1, D2 and D3) and subsampled for pooled carcass composition analyses (*n* = 3 diet^−1^).

	D1	D2	D3	*F*-value	*p*-Value
Mass (g)	46.0 ± 7.1	43.4 ± 4.5	42.9 ± 6.3	*F*_(2,27)_ = 0.726	0.493
Length (cm)	15.3 ± 0.5	15.5 ± 0.4	15.2 ± 0.8	*F*_(2,27)_ = 0.557	0.580
Carcass composition (% dry weight [DW])					
Carcass moisture (%)	71.8 ± 0.2	71.5 ± 0.1	71.9 ± 0.5	*F*_(2,6)_ = 0.934	0.443
Ash (% DW)	10.7 ± 0.4	9.5 ± 0.7	9.6 ± 0.7	*F*_(2,6)_ = 3.199	0.114
Total Lipid (% DW)	19.1 ± 2.7	17.2 ± 0.4	19.0 ± 2.0	*F*_(2,6)_ = 0.902	0.454
Crude protein (% DW)	64.8 ± 6.8	60.1 ± 3.0	57.3 ± 7.3	*F*_(2,6)_ = 1.199	0.365
Gross energy (MJ/kg)	23.7 ± 0.7	23.7 ± 0.2	23.4 ± 0.4	*F*_(2,6)_ = 0.494	0.633

*Note:* Values are in mean ± standard deviation (SD).

**Table 6:** Oxygen uptake rate of Atlantic salmon parr acclimated at 22°C and fed three experimental diets (D1, D2 and D3).

	Diet			*F*_(df num, df res)_	*p*-Value
	D1 (*n* = 10)	D2 (*n* = 13)	D3 (*n* = 11)		
Mass (g)	50.6 ± 8.8	49.9 ± 5.6	50.2 ± 6.2	*F*_(2,31)_ = 0.025	*p* = 0.975
Length (cm)	15.3 ± 1.0	15.4 ± 0.6	15.4 ± 0.7	*F*_(2,31)_ = 0.063	*p* = 0.939
Feed intake (% BM)	0.50 ± 0.02	0.51 ± 0.02	0.52 ± 0.02	*F*_(2,31)_ = 2.098	*p* = 0.140
Energy of the meal ingested (kJ)	6.2 ± 0.9	6.2 ± 0.7	6.4 ± 0.8	*F*_(2,31)_ = 0.248	*p* = 0.782
SMR (mg O_2_ kg^−1^ h ^−1^)	142 ± 9	148 ± 17	143 ± 12	*F*_(2,31)_ = 0.659	*p* = 0.525
MMR (mg O_2_ kg^−1^ h^−1^)	537 ± 48	568 ± 57	574 ± 35	*F*_(2,31)_ = 1.762	*p* = 0.189
AS (mg O_2_ Kg^−1^ h^−1^)	395 ± 50	421 ± 58	431 ± 35	*F*_(2,31)_ = 1.441	*p* = 0.252
SDA_peak_ (mg O_2_ kg^−1^ h^−1^)	207 ± 16	225 ± 20	212 ± 18	*F*_(2,31)_ = 3.254	*p* = 0.052
Peak_net_ (mg O_2_ kg^−1^ h^−1^)	65 ± 16	78 ± 18	69 ± 16	*F*_(2,31)_ = 1.759	*p* = 0.189
*T*_peak_ (h)	5.1 ± 3.4 (3–7.1)	4.0 ± 2.4 (3–10)	6.5 ± 5.6 (3–20.8)	*F*_(2,31)_ = 1.194	*p* = 0.317
SDA magnitude (mg O_2_ Kg^−1^)	1,096 ± 347	1,206 ± 273	1,070 ± 252	*F*_(2,31)_ = 0.923	*p* = 0.408
SDA cost (kJ kg^−1^)	15.4 ± 4.9	16.9 ± 3.8	15.0 ± 3.5	*F*_(2,31)_ = 0.923	*p* = 0.408
SDA duration (h)	26.8 ± 3.5	26.3 ± 4.2	25.5 ± 4.3	*F*_(2,31)_ = 0.242	*p* = 0.787
% AS occupied by Peak_net_	16.3 ± 2.7	18.7 ± 4.2	16.1 ± 3.8	*F*_(2,31)_ = 1.808	*p* = 0.180
SDA scope	1.46 ± 0.12	1.54 ± 0.15	1.49 ± 0.13	*F*_(2,31)_ = 0.849	*p* = 0.438
SDA coefficient (%)	12.5 ± 3.8	13.6 ± 2.7	11.8 ± 3.0	*F*_(2,31)_ = 0.978	*p* = 0.387

*Note:* Values are presented in mean ± SD. *T*_peak_ also has a range between parentheses.

Abbreviations: AS, aerobic scope; BM, body mass; MMR, maximum metabolic rate; SDA, specific dynamic action; SMR, standard metabolic rate.

**Table 7:** Fold (hF) and enterocyte (hE) height (*n* = 7–10 diet^−1^) of Atlantic salmon parr.

	D1	D2	D3	*F*-value	*p*-Value
Pyloric caeca					
hF	207.2 ± 42.5^B^	214.7 ± 40.2^A,B^	236.6 ± 45.2^A^	D: *F*_(2,21.7)_ = 4.245	**0.028**
				M: *F*_(1,19.8)_ = 0.377	0.546

hE	32.7 ± 4.9	31.2 ± 4.8	33.2 ± 4.5	D: *F*_(2,19.9)_ = 3.172	0.064
				M: *F*_(1,22.3)_ = 0.435	0.516

Midintestine					
hF	287.0 ± 70.6	292.9 ± 66.3	313.5 ± 72.7	D: *F*_(2,22.1)_ = 2.303	0.123
				M: *F*_(1,21.7)_ = 5.572	**0.028**

hE	33.7 ± 6.4	32.0 ± 5.8	31.1 ± 4.8	D: *F*_(2,22)_ = 1.727	0.201
				M: *F*_(1,22)_ = 1.022	0.323

*Note:* Values as mean ± standard deviation (SD).

Abbreviations: D, diet; M, mass.

^A,B^Superscript letters of significance indicated *p*  < 0.05. Statistically significant effects are indicated in bold.

## Data Availability

The data that support the findings of this study are available in UQ eSpace at https://doi.org/10.48610/4a2e78e.
